# Case of Erysipelas Neanatorum

**Published:** 1858-04

**Authors:** F. K. Bailey

**Affiliations:** Joliet, Ill.


ARTICLE IV.

### CASE OF ERYSIPELAS NEANATORUM.

BY F. K. BAILEY, M.D., JOLIET, ILL.

Was called on Tuesday, P. M., May 26th, 1857, to see a female infant, six weeks old, which had for two or three days preceding been afflicted with convulsive jerkings of the limbs and muscles of the trunk, with fever, restlessness, and other unfavorable symptoms.

During the forenoon of the same day, the mother had placed the child in a warm bath, and within an hour, its body, from the umbilical region to the pubic, was covered with an erysipelatous eruption, with considerable swelling about the hips and genital region, and increased heat over the whole surface affected.

On my arrival, there was burning fever, rolling of the eyes, and convulsive startings. Pulse frequent, and very feeble; tongue covered with a white coat from the base, to within a half an inch of the lip, (which space was very red), with inflamed and elevated papillæ. The conjunctiva was congested, and the countenance bore every mark of approaching convulsions. The bowels were constipated, stomach irritable, with occasional vomiting, and eructations of an extremely acid nature. Directed warm applications to the inflamed part, with cold to the head. To apply mustard fomentations to the pit of the stomach, and feet; and to place the little patient in a warm bath, should convulsive appearances continue. Prescribing small powders of hyd. cum. creta, pulv. Doveri, and sul. quinia, every two hours.

*Wednesday Morning*, 27*th*. Less fever, and more quiet. Eruption has extended to the knees, and also reached the upper part of the body, but less red than at first, with a marked diminution of heat. To continue powders, with wine and water. To apply mucilage of slippery elm to the inflamed surface, with strict injunctions to keep the head cool.

*Evening*. There has been but little fever through the day, but the stomach more irritable, eruption slowly extending, but less malignant. Free alvine evacuation during the day; convulsive symptoms diminishing. To take pulv. Doveri and sul. quinia every two hours, with wine, through the night.

*Thursday Morning*. Constitutional symptoms relieved. Redness has extended to the feet, with occasional spots upon the face and scalp. Stomach so irritable, as to compel a discontinuance of the powders during the night, but the wine was tolerated. To continue the same course of treatment, if the stomach would retain medicine, and to give bath, one in six or eight hours, in water about 70°.

*Friday Morning*, 28*th*. Found feet and legs much swollen; the fulness about the hip much as at first, and considerable tumefaction around the eyes, and nearly closing those organs. Appearances on the whole rather unfavorable, and apprehensions of a fatal termination increasing. No fever, but the child is very restless, and fretful. Seems to suffer greatly both from the local disease, and from constitutional depression. To continue wine, and to give the powders as before, if the stomach will tolerate them. No more movement of the bowels, nor is anything in that direction desired. The warm bath to be used as occasion may require.

*Saturday Morning*, 29*th*. Still feeble, but the swelling and redness on the hips and parts adjacent subsiding. To continue treatment as directed yesterday.

*Sunday*, 30*th*. General appearances much more favorable. No fever, swelling continues in the feet, but nearly gone from the body. Noticed a small tumor upon the scalp, in the occipital region.

*Monday*, 31*st*. Redness and tumefaction nearly disappeared. No convulsive appearances, and takes the breast as well as ever.

*Friday*, *June* 4*th*. Desquamation commencing. The swelling upon scalp subsiding. To take sul. quinia in small doses three time daily.

*Tuesday*, 8*th*. The original morbid appearances have entirely subsided. The left knee, however, is much swollen, and the tendons in the poplitœal space contracted. The joint so painful as to make motion impossible. There is also a lump two inches below the patella of the same limb, upon the anterior face of the tibia, and appearing much as that upon the head. Applied diluted tincture iodine to the tumor, and directed poultices, with a little tr. opii added, to be applied to the knee. To continue quinia, and to take small doses of comp. syr. sarsaparilla, in which a few grains of sod. potass. had been dissolved, three times daily.

*June* 17*th*. Made free incision in the knee, and pus freely escaped. The child has improved in health since last report, notwithstanding the suppurative process going on in the knee. From this period, nothing occurred to impede recovery, and in a short time the child was entirely well.

The disease of which the above case is an instance, is mentioned by many writers, and all concur in its being rare, and generally fatal. The fact of its rare occurrence, and the ultimate favorable issue of the case, rendered it deeply interesting to me; and so much so, that I decided to send a copy of my notes to the *Journal*. Before closing, it may not be amiss to mention one or two facts not noted as they occurred, but which may not be uninteresting. The mother, though apparently enjoying usual health, is rather delicate, and subsequent to the birth of the child, had occasional attacks of ague. To combat this condition, she had taken nothing until after the child was taken sick, but some homœopathic medicines, which will be generally considered equivalent to doing nothing at all or neglect of interfering with disease. As she secreted a liberal quantity of milk, and the child had depended upon that source for nourishment, and was still nursing, when able to do so, it occurred to me that a liberal diet, with brandy punch taken as freely as the stomach and head would bear, was indicated in the case of the mother. The above course was adopted, and to all appearances should claim a good share of the credit in the cure of the child. The mental and physical condition of the mother while nursing an infant, should always receive attention, and especially while the infant is laboring under a severe constitutional disease.

A woman of nervous temperament, and whose feelings and sympathies are excited to such a degree as both to deprive her of appetite for food, and ability to sleep, must communicate a very depressing influence to the little being who derives its only sustenance from her breast.

I have for years been in the habit of directing attention to the condition of the mother under circumstances such as are above mentioned. Dr. Condie, in his work upon disease of children, has referred to this, and others have also mentioned the importance of attention in this direction; still, practitioners are not sufficiently aware of these demands upon their notice. The state of the mind is of equal, if not greater, importance than that of the physical organization, because grief, anxiety, and suspense, will influence the secretion, not only of milk, but that of many other organs. These remarks will apply in cases of cholera infantum, diarrhœa, and other exhausting diseases of children, when much depends upon the kind and quantity of nourishment.

